# Highly pathogenic avian influenza H7N9 viruses with reduced susceptibility to neuraminidase inhibitors showed comparable replication capacity to their sensitive counterparts

**DOI:** 10.1186/s12985-019-1194-9

**Published:** 2019-07-02

**Authors:** Jing Tang, Jing Zhang, Jianfang Zhou, Wenfei Zhu, Lei Yang, Shumei Zou, Hejiang Wei, Li Xin, Weijuan Huang, Xiyan Li, Yanhui Cheng, Dayan Wang

**Affiliations:** National Institute for Viral Disease Control and Prevention Chinese Centers for Disease Control and Prevention Key Laboratory for Medical Virology, National Health Commission, NO.155 Changbai road, Changping District, Beijing, 102206 People’s Republic of China

**Keywords:** Influenza virus, Highly pathogenic avian influenza H7N9, Neuraminidase, Drug resistance, Replication capacity, Reverse genetics

## Abstract

**Background:**

Human infection with avian influenza H7N9 virus was first reported in 2013. Since the fifth epidemic, a highly pathogenic avian influenza (HPAI) H7N9 virus has emerged and caused 33 human infections. Several potential NAI resistance sites have been found in human cases. However, the drug susceptibility and replication ability of HPAI H7N9 virus with such substitutions have not yet been studied.

**Methods:**

Thirty-three HPAI H7N9 virus strains were isolated from human cases in China, and then sequences were analyzed to identify potential NAI resistance sites. Recombinant influenza viruses were generated to evaluate the effect of NA amino acid substitutions on NAI (oseltamivir or zanamivir) susceptibility and viral replication efficiency in MDCK cells.

**Results:**

Four potential NAI resistance sites, R292 K, E119V, A246T or H274Y, were screened. All four substitutions conferred either reduced or highly reduced susceptibility to oseltamivir or zanamivir. 292 K not only highly reduced the susceptibility of HPAI H7N9 to oseltamivir but also induced an increase in the IC50 of zanamivir. 119 V or 274Y conferred reduced susceptibility of HPAI H7N9 to oseltamivir. Additionally, 246 T conferred reduced susceptibility to zanamivir. All tested NAI-resistant viruses were capable of replication in MDCK cells. The virus yields of rg006-NA292K were lower than those of rg006-NA292R at 24, 48, 72 and 96 h postinfection (*P*<0.05). Rg006-NA119V, rg006-NA246T or rg006-NA274Y showed comparable replication capacity to wild-type virus (except for rg006-NA274Y at 96 h, P<0.05).

**Conclusions:**

All 4 amino acid substitutions (R292 K, E119V, A246T or H274Y) in NA reduced the susceptibility of HPAI H7N9 to NAIs. The NAI-resistant mutations in HPAI H7N9, in most cases, did not reduce the replication ability of the virus in mammalian cells. Special attention needs to be paid to these mutations, and the development of new anti-H7N9 drugs is of great importance.

**Electronic supplementary material:**

The online version of this article (10.1186/s12985-019-1194-9) contains supplementary material, which is available to authorized users.

## Background

In March 2013, China reported the first human infection with a novel reassortant avian influenza H7N9 virus [1]. As of 27 April 2019, the avian influenza A (H7N9) virus has caused five significant epidemic waves, leading to 1567 laboratory-confirmed cases and 614 fatal cases (http://www.chinaivdc.cn/cnic/en/Surveillance/WeeklyReport/201904/t20190427_201686.htm). The number of H7N9 human cases was greater than that of any other avian influenza virus that infects humans. The viruses detected in the first four waves were all low pathogenic avian influenza (LPAI) H7N9 viruses, which were nonlethal in poultry. The fifth epidemic wave started from Oct. 1, 2016 to Sep. 30, 2017, with 779 laboratory-confirmed cases reported in mainland China, and it is considered the largest epidemic wave so far [2]. Multiple basic amino acid insertions in the cleavage site of hemagglutinin have been observed since the fifth wave, which supported the emergence of HPAI H7N9 viruses [3–5]. HPAI H7N9 viruses, which are lethal in chickens, were also detected in poultry in wave five [6]. In addition, HPAI H7N9 viruses have evolved rapidly, and some strains have become well adapted to and lethal in ducks [7]. Human and poultry infections by HPAI H7N9 viruses across China pose a great challenge to disease prevention and control.

Since 2010, neuraminidase inhibitors (NAIs) are the only class of antivirals recommended by the WHO for the treatment of influenza infections [8]. The approved NAIs in China, including oseltamivir, zanamivir and peramivir, are currently the principal treatment options for managing influenza infection [9]. There are 19 highly conserved residues in the NA active site of all influenza A and B viruses. These include eight catalytic residues (R118, D151, R152, R224, E276, R292, R371, and Y406) that directly contact the sialic acid (SA) and 11 framework residues (E119, R156, W178, S179, D198, I222, E227, H274, E277, N294, and E425) that support the enzymatic binding pocket [10]. When amino acid mutations occur in key regions of NA, the sensitivity of the virus to NAIs may be reduced, thus affecting the therapeutic effect of the drugs.

We previously reported that the proportion of viruses with mutations potentially related to reduced susceptibility to NAIs in LPAI H7N9 was approximately 6% [11], while more than 20% in HPAI H7N9 [5]. One study reported the introduction of 14 amino acid substitutions in the recombinant neuraminidase protein of A/Shanghai/2/2013 (LPAI H7N9) for drug susceptibility evaluation. The research demonstrated that R292K and H274Y conferred a high increase in oseltamivir IC_50_, and E119D conferred the highest IC_50_ to zanamivir reported [12]. It was recently reported that artificially created HPAI H7N9 with an NA 294 K mutation caused multidrug resistance and attenuated virus replication in NHBE cells [13]. However, systematic identification of potential NAI resistance sites and growth characteristics of HPAI H7N9 viruses isolated from human cases have not yet been reported.

In this study, recombinant influenza viruses were generated in the homogeneous genetic background of the HPAI H7N9 virus, A/Guangdong/17SF006/2017 (H7N9). NA substitutions associated with reduced inhibition to NAIs, which were found in HPAI H7N9 viruses isolated from patients, were introduced into A/Guangdong/17SF006/2017 (H7N9) by reverse genetics. We examined the effect of NA amino acid substitutions on NAI susceptibility and viral replication efficiency in vitro.

## Methods

### Potential NAI resistance mutations in HPAI H7N9 influenza viruses from human cases

Since the fifth epidemic wave, a highly pathogenic avian influenza (HPAI) H7N9 virus has emerged and caused human infections in China. HPAI H7N9 viruses isolated from human cases collected from Dec. 1, 2016, to Apr. 27, 2019, were obtained from the Chinese National Influenza Surveillance Network, which covers 32 provinces in mainland China (including the autonomous regions/municipalities directly under the Central Government), including 408 network laboratories and 554 sentinel hospitals. Virus isolation was conducted in a biosafety level 3 facility by inoculating 0.2-ml original samples allantoically into 9~11-day-old specific-pathogen-free (SPF) embryonated chicken eggs. After sequencing, HPAI H7N9 sequences were analyzed to identify amino acid substitutions related to potentially reduced susceptibility to NAIs. The criteria for screening potential drug resistance sites of HPAI H7N9 virus were as follows: 1) NAI resistance sites in other subtypes of influenza virus that have been reported previously and 2) 19 highly conserved residues in the NA active site of all influenza A, which may be related to resistance.

### Cell preparation

Madin-Darby canine kidney (MDCK) cells and human embryonic kidney (293 T) cells were grown and maintained in Dulbecco’s modified Eagle’s medium (DMEM; Invitrogen) supplemented with 10% fetal bovine serum (FBS; Invitrogen), HEPES (10 mM, Invitrogen), penicillin (100 units/ml), and streptomycin (100 μg/ml, Invitrogen).

### Site-directed mutagenesis

A virus containing amino acid substitutions in sites related to potentially reduced susceptibility to NAIs in HPAI H7N9 viruses, including R292 K, E119V, A246T and H274Y, was constructed in this study. An HPAI H7N9 virus, A/Guangdong/17SF006/2017 isolated from humans that had resulted in fatal outcomes, was used as the backbone virus, and its NA contained 292 K. All eight gene segments of influenza virus A/Guangdong/17SF006/2017 (H7N9) were artificially synthesized (Sangon Biotech) and cloned into the pHW2000 vector. The wild-type A/Guangdong/17SF006/2017 (H7N9) virus encoded 292 K, 119E, 246A and 274H in the NA protein. First, a single-point mutation was introduced to convert 292 K in NA to 292R. Then, in the 292R background, single-point mutations 119 V, 246 T or 274Y in the NA gene were carried out. 292R, 119 V, 246 T or 274Y substitutions were generated with the following primers:

- NA K292R Forward, ccctgccaattgtccctgcatgtgcaggtaatc;
- NA K292R Reverse, gattacctgcacatgcagggacaattggcaggg;
- NA E119V Forward, gtcgcatgaaacatagggtactcttgtgactaaaacatc;
- NA E119V Reverse, gatgttttagtcacaagagtaccctatgtttcatgcgac;
- NA A246T Forward, caccgatgggcctacaactggacctgcag;
- NA A246T Reverse, gtggctacccggatgttgacctggacgtc;
- NA H274Y Forward, ggaactgctaagtacattgaagaatg;
- NA H274Y Reverse, ccttgacgattcatgtaacttcttac.

Mutations were introduced into the NA plasmid using the QuikChange Site-Directed Mutagenesis Kit (Stratagene). The presence of the introduced mutations and the absence of additional unwanted mutations were verified by sequencing of the entire cDNA.

### Generation of recombinant viruses and virus titration

The recombinant viruses were generated by reverse genetics as previously reported. In general, the eight gene segments were cloned into the pHW2000 vector and cotransfected into 293 T/MDCK cocultured monolayers at 37 °C for 24 h [14]. Then, Opti-MEM supplemented with trypsin (1 μg/ml) (Sigma-Aldrich) was added. Culture supernatant was collected at 72 h and inoculated into 9~11-day-old SPF embryonated chicken eggs. A hemagglutination test was used for the detection of productive virus infection. Virus stocks were sequenced for verification, and virus titers were determined on MDCK cells.

### NA inhibition test

The NA inhibitors oseltamivir carboxylate (oseltamivir; Hoffman-La Roche) and zanamivir (GlaxoSmithKline) were prepared in sterile distilled water and stored in aliquots at − 30 °C until use.

Inhibition of NA enzyme activity by the 2 NA inhibitors was assessed in the fluorescence-based NA inhibition assay using the NA-Fluor Influenza Neuraminidase Assay Kit (Applied Biosystems, ThermoFisher) as previously described [15]. IC_50_ values, defined as the concentration of drug required to reduce enzyme activity by 50%, were calculated using GraphPad Prism 5 software. The IC_50_ values reported are the means (±SD) of IC_50_ values measured for at least 2 independent tests with 2 duplicates for each sample. Interpretation of IC_50_ values (obtained by comparing the test virus with the drug-sensitive reference virus) was performed using the WHO AVWG criteria for influenza A viruses: a < 10-fold increase in IC_50_ represents normal inhibition, a 10~100-fold increase represents reduced inhibition, while a > 100-fold increase is highly reduced inhibition.

### Growth kinetics in MDCK cells

MDCK cells were infected at an MOI of 0.001 with the recombinant viruses for multicycle replication under the conditions of 5% CO_2_ and 37 °C. After 1 h of incubation, cells were washed twice with PBS, and infection medium containing TPCK-trypsin was added. At time points 0, 18, 24, 48, 72 and 96 h post-infection, supernatants were collected, and 3 biological repeats were set up for each sample. Viral titers were determined on MDCK cells.

### Crystal structure display of NA resistance sites

The existing NA protein crystal structure of A/Anhui/1/2013 H7N9 virus (PDB ID: 4MWJ) in Protein Data Bank (PDB) was used, based on which a crystal structure model of the NA monomer was obtained. DeepView v4.1.0 software was used to display and label drug resistance-related mutations.

### Statistical analysis

Student’s t test (unpaired, two-tailed) was calculated using GraphPad Prism software (GraphPad Software Inc., CA, USA). A *P* value of < 0.05 was considered significant.

### Biosafety

All experiments involving HPAI H7N9 viruses were performed by qualified personnel in a BSL-3 laboratory.

## Results

### Potential NAI resistance mutations in HPAI H7N9 viruses from human cases

Up until April 2019, 33 cases were confirmed to be infected with HPAI H7N9 virus based on the sequence of hemagglutinin, with illness onset dates from December 2016 to April 2019. Among them, 15 were fatal cases. Four potential NAI resistance sites, 292 K, 119 V, 246 T or 274Y in the NA protein, have been identified in HPAI H7N9-infected cases (Table 1), and the characterization of other viral proteins is shown in Additional file 1: Table S1. In total, 9 human cases were infected with HPAI H7N9 viruses containing potential NA resistance mutations (9/33, 27%), including 5 fatal cases and 4 recovered cases (Table 1).

**Table 1:** Potential NAI resistance mutations in HPAI H7N9 virus from human cases

Protein	Site	Function	Amino Acid	Human Cases
NA	115^a^(119^b^)	Potential reducing the susceptibility of neuraminidase inhibitors	E(GAA)	31
			V(GTA)	1
	243^a^ (246^b^)		A(GCC)	31
			T(ACA)	1(fatal case)
	271^a^ (274^b^)		H(CAT)	31
			Y(TAT)	1(fatal case)
	289^a^ (292^b^)		R(AGG)	26
			R\K	1(fatal case)
			K(AAG)	5(2fatalcases)

In China, NAI_S_ (oseltamivir, zanamivir or peramivir) are used in hospitalized cases for influenza treatment, of which oseltamivir is the most common [16]. Among the 9 human cases infected with HPAI H7N9 viruses containing the above potential NA resistance mutations, 7 cases were treated with NAIs. The use of NAIs by the other 2 patients is unknown.

### Generation of a recombinant, highly pathogenic avian influenza a (H7N9) virus

In this study, A/Guangdong/17SF006/2017 (H7N9) was used as a backbone (called rg006-NA292K), which contained NA 292 K – a mutation related to reduced susceptibility to NAIs. A NAI-sensitive virus containing NA 292R, called rg006-NA292R, was constructed first by reverse genetics based on rg006-NA292K. The other 3 amino acid substitutions (119 V, 246 T, 274Y) were introduced into the cDNA encoding the NA of rg006-NA292R. The recombinant viruses (called rg006-NA119V, rg006-NA246T and rg006-NA274Y) were generated by reverse genetics. The nucleotide changes were as follows: for E119V, E/GAA to V/GTA; for A246T, A/GCC to T/ACA; for H274Y, H/CAT to Y/TAT (Table 1). All the constructed plasmids were sequenced to ensure fidelity to the strain and/or the presence of the desired mutations. All rescued viruses had sufficient NA activity (data not shown) for NI assay testing.

### Assessment of susceptibility to neuraminidase inhibitors

The NAI assay was used to investigate the effect of the introduced amino acid substitutions on drug susceptibility. The resulting NA inhibition profiles for oseltamivir and zanamivir are shown in Table 2. A/Texas/12/2007 (H3N2 119E) and A/Texas/12/2007 (H3N2 119 V) were used as control viruses in the drug sensitivity test. The E119V mutation in N2 has been reported to significantly reduce the sensitivity of the virus to oseltamivir but not to zanamivir. The data in this study were consistent with previous reports. Rg006-NA292R virus, which encodes 119E, 246A and 274H in the NA protein, is sensitive to oseltamivir (mean IC_50_ (nM) ± SD, 1.24 ± 0.01) or zanamivir (mean IC_50_ (nM) ± SD, 1.50 ± 0.10). However, the substitution E119V in the NA protein induced a mean 90.77-fold increase in the IC_50_ of oseltamivir (mean IC_50_ (nM) ± SD, 112.55 ± 17.25). In addition, the substitution H274Y in the NA protein induced a mean 23.40-fold increase in the IC_50_ of oseltamivir (mean IC_50_ (nM) ± SD, 29.01 ± 0.18). Furthermore, the substitution A246T in the NA protein induced a mean 10.97-fold increase in the IC_50_ of zanamivir (mean IC_50_ (nM) ± SD, 16.46 ± 0.59). The 292 K in the NA protein induced a mean 3686.29-fold increase in the IC_50_ of oseltamivir (mean IC_50_ (nM) ± SD, 4571 ± 54.7) and a mean 21.68-fold increase in the IC_50_ of zanamivir (mean IC_50_ (nM) ± SD, 32.52 ± 4.53). 292 K in the NA protein of HPAI H7N9 virus induced high and multiple-drug resistance. In conclusion, the reverse genetically constructed HPAI H7N9 viruses containing 292 K, 119 V, 274Y or 246 T in NA could reduce the sensitivity of viruses to oseltamivir or zanamivir.

**Table 2:** Susceptibility of highly pathogenic avian influenza rg006 H7N9 viruses to neuraminidase inhibitors

Viruses	Oseltamivir		Zanamivir	
	Mean IC_50_^a^(nM) ± SD	Fold change^b^	Mean IC_50_^a^(nM) ± SD	Fold change^b^
rg006-NA292R	1.24 ± 0.01	1	1.5 ± 0.10	1
rg006-NA119V	112.55 ± 17.25	90.77^c^	8.39 ± 0.44	5.59
rg006-NA292K	4571 ± 54.7	3686.29^c^	32.52 ± 4.35	21.68^c^
rg006-NA274Y	29.01 ± 0.18	23.40^c^	1.76 ± 0.13	1.17
rg006-NA246T	1.85 ± 0.11	1.49	16.46 ± 0.59	10.97^c^
A/Texas/12/2007 (H3N2 119E)^d^	0.12 ± 0.01	1	1.71 ± 0.63	1
A/Texas/12/2007 (H3N2 119 V)^d^	62.41 ± 2.16	520.08^c^	1.56 ± 0.55	0.91

^a^IC_50_, half-maximal inhibitory concentration. The IC_50_ denotes the concentration of a NA inhibitor that reduces the NA activity by 50% relative to NA activity without the inhibitor

^b^Fold change relative to the mean IC_50_ of the wild-type NA protein. Fold-change values of each NA were interpreted using criteria established by the World Health Organization Influenza Antiviral Working Group

^c^Fold change > 10 (including reduced/highly reduced inhibition)

^d^A/Texas/12/2007 (H3N2 119E) and A/Texas/12/2007 (H3N2 119 V), control virus

### Growth characteristics of mutant viruses

**Fig. 1:**
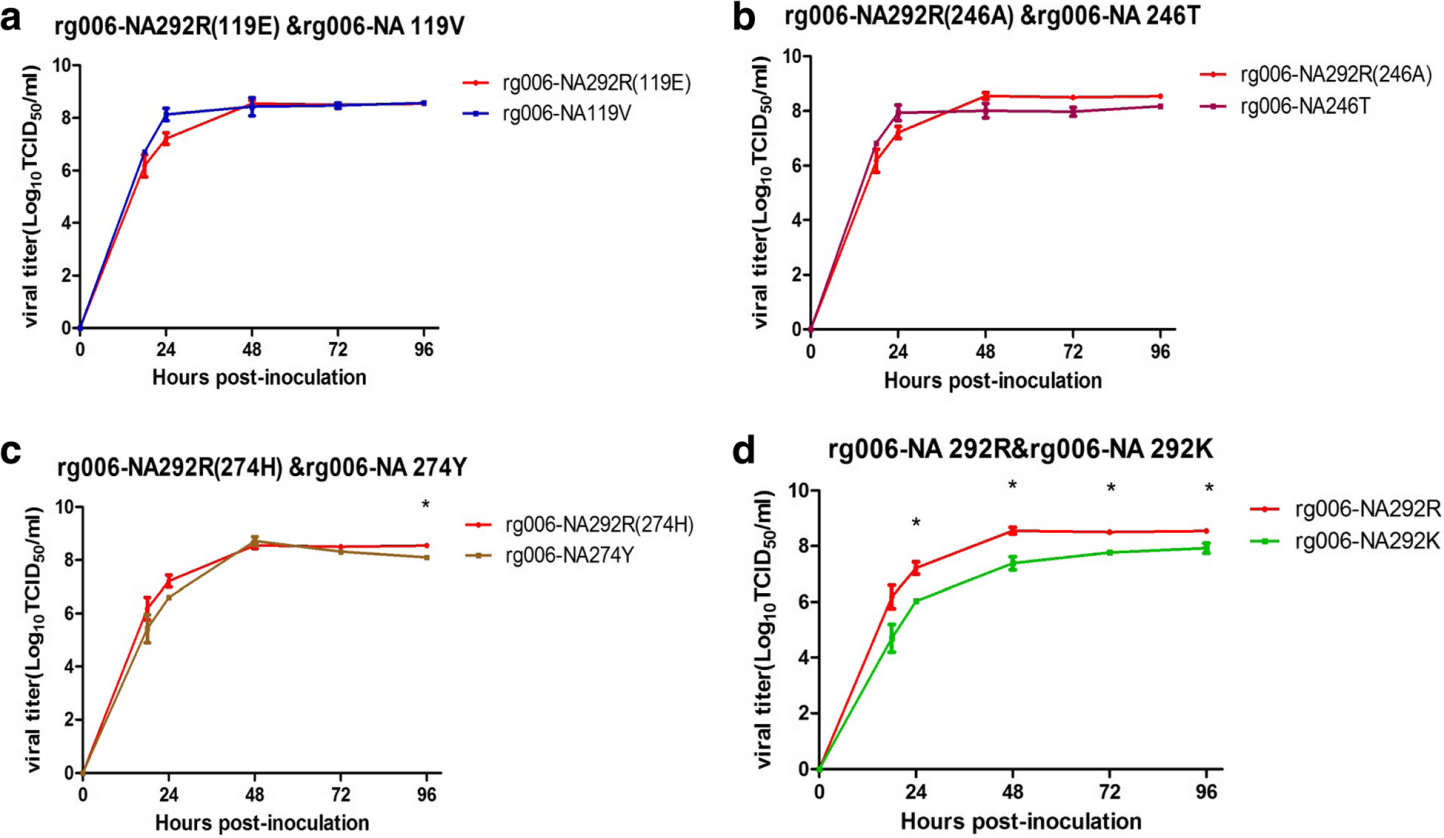
Replication kinetics of recombinant viruses in MDCK cells. Cells were inoculated with the recombinant viruses at 37 °C, and culture supernatants were harvested at 0, 18, 24, 48, 72 and 96 h post-inoculation. Virus titers were determined on MDCK cells. Growth curve of the rg006-NA292R(119E, 246A, 274H) in MDCK cells was in red, round; rg006-NA119V viruses were in blue, square (**a**); rg006-NA246T virus in purple, square (**b**); rg006-NA274Y virus in brown square (**c**); rg006-NA292Kviruses in green, square (**d**). Each data point represents the log10 mean ± SD TCID_50_/mL from at least two independent tests. *P < 0.05

To test the effect of the NAI resistance mutations on the growth characteristics of the virus, we determined infectious titers in MDCK cells. MDCK cells were infected at an MOI of 0.001 with the recombinant viruses to allow multicycle replication. At time points 0, 18, 24, 48, 72 and 96 h post-infection, viral titers were determined on MDCK cells. Virus kinetics experiments showed that the rg006-NA119V, rg006-NA246T and rg006-NA274Y viruses have similar growth kinetics to viruses containing 119E, 246A and 274H in MDCK cells (Fig. 1). No significant difference in infectious titers in MDCK cells was observed (except for rg006-NA274Y at 96 h, P<0.05). This implies that the mutations studied did not have much of an effect on the growth characteristics of the viruses in MDCK cells, even if some mutations strongly decreased the NA activity of the virus (data not shown). The yields of rg006-NA292K were significantly lower than those of rg006-NA292R at 24, 48, 72 and 96 h (P<0.05). However, rg006-NA292K still maintains a considerable level of replication in MDCK cells. Overall, all tested NAI-resistant viruses were capable of replicating in MDCK cells. Rg006-NA119V, rg006-NA246T and rg006-NA276Y showed comparable replication capacity to their sensitive counterparts.

## Discussion

Before the fifth epidemic wave, the H7N9 virus that had been causing human infections since 2013 was weakly pathogenic to birds, although it was highly pathogenic to humans. However, in the fifth wave, which began in October 2016, HPAI H7N9 viruses were detected. Since then, 33 highly pathogenic H7N9-infected cases have been reported, accounting for less than 4% of all cases. Four NA mutations showed reduced susceptibility to NAIs, and R292 K, E119V, A246T and H274Y were identified in highly pathogenic H7N9 viruses from infected cases. In addition, the proportion of viruses with these mutations in highly pathogenic H7N9 was 27%, as reported in this study, which was significantly higher than in LPAI H7N9 viruses.

To avoid the potential effect of quasi species in wild-type virus on susceptibility to NAIs, we constructed viruses with reverse genetics and made directed point mutations at specific NA loci. Studies with the viruses constructed by reverse genetics technology, which excluded interference from other sites, can identify the susceptibility of NA protein to NAIs and evaluate the growth characteristics of the mutant viruses.

As shown in Table 2, all four substitutions conferred either reduced or highly reduced inhibition to oseltamivir or zanamivir. 292 K in the NA protein of HPAI H7N9 virus induced a high and multiple-drug resistance, which induced a mean 3686.29-fold increase in the IC_50_ of oseltamivir and a mean 21.68-fold increase in the IC_50_ of zanamivir. It has been reported that the NA R292K substitution also decreased the sensitivity of HPAI H7N9 to peramivir [4]. 292 K in the NA protein of LPAI H7N9 virus induced a more than 1000-fold increase in the IC_50_ of oseltamivir and a 67-fold increase in the IC_50_ of zanamivir [12, 17, 18]. Because the 292 K mutation showed reduced susceptibility to multiple NAIs and the degree of resistance it conferred was the most notable, especially for oseltamivir, it is suggested that once the 292 K mutation is encountered in H7N9 clinical cases, alternative drugs should be considered.

Single drug resistance to oseltamivir or zanamivir to varying degrees was observed in NA E119V, H274Y or A246T of HPAI H7N9 virus. The increases in the IC_50_ of oseltamivir conferred by E119V and H274Y were 90.77-fold and 23.40-fold, respectively. Additionally, A246T conferred reduced inhibition by zanamivir. Regarding LPAI H7N9, the increase in the IC_50_ of oseltamivir conferred by E119V and H274Y were 169-fold and 812-fold [12], respectively. In conclusion, with the emergence of amino acid substitutions at key sites within NA, both LPAI and HPAI H7N9 viruses decreased their susceptibility to NAIs.

**Fig. 2:**
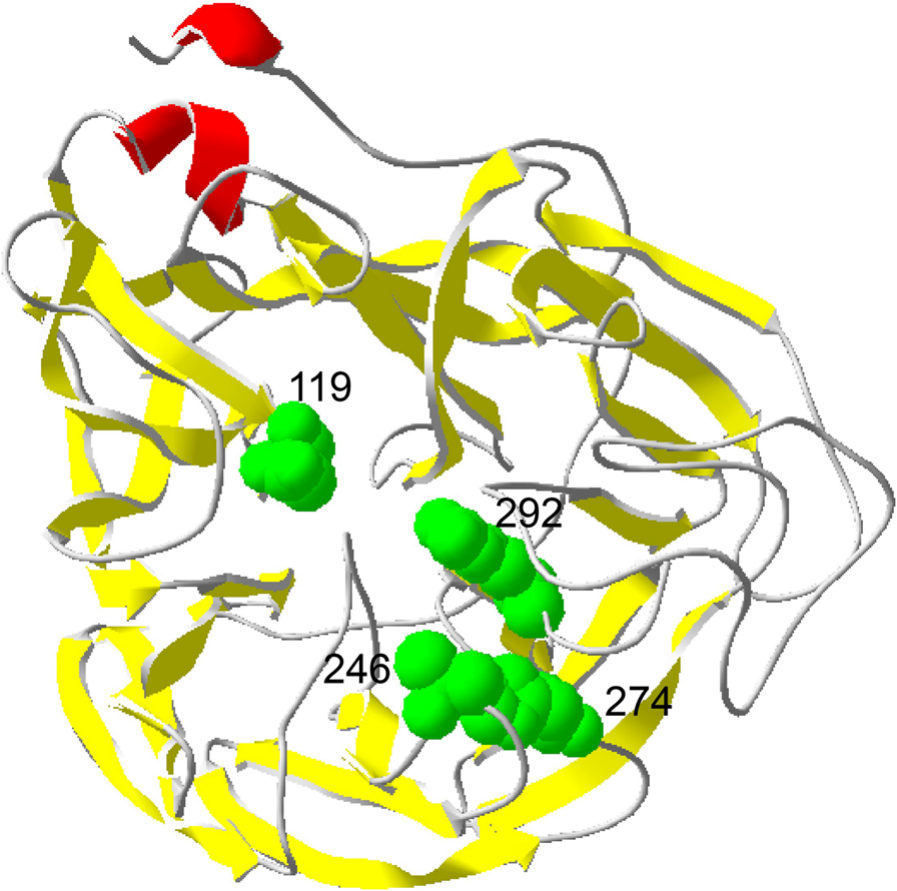
Crystal structure display of the four neuraminidase drug resistance sites of human infection with highly pathogenic avian influenza H7N9 virus. Crystal structure model of NA monomer of A/ Anhui1/2013/H7N9 virus (PDB ID: 4MWJ) was used to display and label the four drug resistance mutations (292 K, E119V, A246T or H274Y) by DeepView v4.1.0 software. The mutations associated with drug resistance in NA were showed in green font

Based on our study, one of the reasons for the higher proportion of strains with reduced susceptibility to NAIs in HPAI H7N9 avian influenza viruses may be their replication capacity in mammalian cells. Generally, when influenza viruses undergo drug-resistant mutations, their replication level in cells or their pathogenicity to animals may be reduced to varying degrees, especially when mutations occur in catalytic residues (R118, D151, R152, R224, E276, R292, R371, and Y406) that directly contact the sialic acid (SA) [19, 20]. As shown in Fig. 2, a crystal structure model of the NA monomer of the A/Anhui/1/2013 H7N9 virus (PDB ID: 4MWJ) was used to display and label the four drug resistance mutations (292 K, E119V, A246T or H274Y) in the HPAI H7N9 virus. 292 K formed the wall of the catalytic pocket, which interacts directly with the substrate, sialic acid. 119 V, 246 T and 274Y are framework sites that interact with catalytic residues to stabilize the active site. However, our study showed that the replication capacity of rg006-NA119V, rg006-NA246T or rg006-NA274Y on MDCK cells was comparable to that of sensitive strains. Although the yields of rg006-NA292K were significantly lower than those of rg006-NA292R at 24, 48, 72 and 96 h postinfection (P<0.05), they still maintained a relatively good level of replication in MDCK cells. Compensatory mutations in some segments of the influenza virus might contribute to the maintenance of the growth characteristics of the H7N9 virus.

292 K, which frequently occurs in the HPAI H7N9 drug-resistant strain, deserves special attention. 292 K has also been found in a chicken-derived HPAI H7N9 strain in LPM during wave 5 [21]. Usually, amino acid substitutions in NA conferring NAI resistance are found mainly in clinics after treatments with NAIs [3, 22]. Seven of the 9 human cases infected with HPAI H7N9 virus containing NA resistance mutations had clear information on receiving NAI treatment, with 6 cases treated before or on the same day as sample collection during their hospitalization. The emergence of HPAI H7N9 resistant mutants may be related to the use of NAIs.

Here, we identified all four potential NAI resistance sites of the HPAI H7N9 virus isolated from human cases in China. The effect of NAI-resistant sites in the HPAI H7N9 virus from human cases on the growth characteristics of the virus was studied for the first time. Our study showed that the occurrence of most mutations did not significantly reduce the growth of the virus in mammalian cells. These NA mutations have been found among fatal cases, so we need to pay special attention to them. Close monitoring of the NA mutations needs to be improved, and it is essential to promote the development and use of alternative antiviral drugs.

## Conclusions

Four potential resistant sites that may reduce the susceptibility of influenza virus to NAIs have been screened from the HPAI H7N9 virus in China. The proportion of potential drug-resistant strains was 27%. As a result, all 4 amino acid substitutions (R292 K, E119V, A246T or H274Y) in the NA protein reduced the susceptibility of HPAI H7N9 to oseltamivir or zanamivir. Most noteworthy, the presence of a NAI resistance mutation in HPAI H7N9, which is highly pathogenic to humans and poultry, in most cases did not reduce the replication ability of the virus in MDCK cells (and was possibly related to the death of some patients). Special attention needs to be paid to these mutations, and the development of new anti-H7N9 drugs is of great importance.

## Additional file

Additional file 1: **Table S1.** Mutation on key sites of HPAI H7N9 virus from human cases. (DOCX 26 kb)

## Data Availability

Not applicable.

## Abbreviations

BSA: Albumin from bovine serum
cDNA: Complementary DNA
DMEM: Dulbecco’s modified eagle medium
FBS: Fetal bovine serum
HPAI H7N9: Highly pathogenic avian influenza H7N9
IC_50_: Half-maximal inhibitory concentration
LPAI H7N9: Low pathogenic avian influenza H7N9
LPMs: Live poultry markets
MDCK: Madin Darby canine kidney
MOI: Multiplicity of infection
NA: Neuraminidase
NAIs: Neuraminidase inhibitors
PBS: Phosphate buffer saline
RG: Reverse genetic

## References

[1] Gao R, Cao B, Hu Y, Feng Z, Wang D, Hu W (2013). Human infection with a novel avian-origin influenza a (H7N9) virus. N Engl J Med.

[2] Wang X, Jiang H, Wu P, Uyeki TM, Feng L, Lai S (2017). Epidemiology of avian influenza a H7N9 virus in human beings across five epidemics in mainland China, 2013–17: an epidemiological study of laboratory-confirmed case series. Lancet Infect Dis.

[3] Zhang F, Bi Y, Wang J, Wong G, Shi W, Hu F (2017). Human infections with recently-emerging highly pathogenic H7N9 avian influenza virus in China. J Inf Secur.

[4] Zhu W, Zhou J, Li Z, Yang L, Li X, Huang W, et al. Biological characterisation of the emerged highly pathogenic avian influenza (HPAI) A(H7N9) viruses in humans, in mainland China, 2016 to 2017. Eurosurveillance. 2017;22(19):30533. 10.2807/1560-7917.ES.2017.22.19.30533 PMC5476987 28537546

[5] Yang L, Zhu W, Li X, Chen M, Wu J, Yu P, et al. Genesis and spread of newly emerged highly pathogenic H7N9 avian viruses in mainland China. J Virol. 2017;91(23):e01277-17. 10.1128/JVI.01277-17 PMC5686710 28956760

[6] Shi J, Deng G, Kong H, Gu C, Ma S, Yin X (2017). H7N9 virulent mutants detected in chickens in China pose an increased threat to humans. Cell Res.

[7] Shi J, Deng G, Ma S, Zeng X, Yin X, Li M (2018). Rapid Evolution of H7N9 Highly Pathogenic Viruses that Emerged in China in 2017. Cell Host Microbe.

[8] Samson M, Pizzorno A, Abed Y, Boivin G (2013). Influenza virus resistance to neuraminidase inhibitors. Antivir Res.

[9] Okomo-Adhiambo M, Sheu TG, Gubareva LV (2013). Assays for monitoring susceptibility of influenza viruses to neuraminidase inhibitors. Influenza Other Respir Viruses.

[10] Burnham AJ, Baranovich T, Marathe BM, Armstrong J, Webster RG, Govorkova EA (2014). Fitness costs for influenza B viruses carrying neuraminidase inhibitor-resistant substitutions: underscoring the importance of E119A and H274Y. Antimicrob Agents Chemother.

[11] Wang D, Yang L, Zhu W, Zhang Y, Zou S, Bo H (2016). Two outbreak sources of influenza a (H7N9) viruses have been established in China. J Virol.

[12] Gubareva LV, Sleeman K, Guo Z, Yang H, Hodges E, Davis CT (2017). Drug susceptibility evaluation of an influenza a(H7N9) virus by analyzing recombinant neuraminidase proteins. J Infect Dis.

[13] Imai M, Watanabe T, Kiso M, Nakajima N, Yamayoshi S, Iwatsuki-Horimoto K (2017). A highly pathogenic avian H7N9 influenza virus isolated from a human is lethal in some ferrets infected via respiratory droplets. Cell Host Microbe.

[14] Fodor E, Devenish L, Engelhardt OG, Palese P, Brownlee GG, Garcia-Sastre A (1999). Rescue of influenza a virus from recombinant DNA. J Virol.

[15] Okomo-Adhiambo M, Nguyen HT, Abd Elal A, Sleeman K, Fry AM, Gubareva LV (2014). Drug susceptibility surveillance of influenza viruses circulating in the United States in 2011-2012: application of the WHO antiviral working group criteria. Influenza Other Respir Viruses.

[16] Zhou L, Tan Y, Kang M, Liu F, Ren R, Wang Y (2017). Preliminary epidemiology of human infections with highly pathogenic avian influenza a(H7N9) virus, China, 2017. Emerg Infect Dis.

[17] Itoh Y, Shichinohe S, Nakayama M, Igarashi M, Ishii A, Ishigaki H (2015). Emergence of H7N9 influenza a virus resistant to neuraminidase inhibitors in nonhuman primates. Antimicrob Agents Chemother.

[18] Zhang X, Song Z, He J, Yen HL, Li J, Zhu Z (2014). Drug susceptibility profile and pathogenicity of H7N9 influenza virus (Anhui1 lineage) with R292K substitution. Emerg Microbes Infect.

[19] Colman PM, Varghese JN, Laver WG (1983). Structure of the catalytic and antigenic sites in influenza virus neuraminidase. Nature..

[20] Colman PM, Hoyne PA, Lawrence MC (1993). Sequence and structure alignment of paramyxovirus hemagglutinin-neuraminidase with influenza virus neuraminidase. J Virol.

[21] Quan Chuansong, Shi Weifeng, Yang Yang, Yang Yongchun, Liu Xiaoqing, Xu Wen, Li Hong, Li Juan, Wang Qianli, Tong Zhou, Wong Gary, Zhang Cheng, Ma Sufang, Ma Zhenghai, Fu Guanghua, Zhang Zewu, Huang Yu, Song Houhui, Yang Liuqing, Liu William J., Liu Yingxia, Liu Wenjun, Gao George F., Bi Yuhai (2018). New Threats from H7N9 Influenza Virus: Spread and Evolution of High- and Low-Pathogenicity Variants with High Genomic Diversity in Wave Five. Journal of Virology.

[22] Wu Y, Bi Y, Vavricka CJ, Sun X, Zhang Y, Gao F (2013). Characterization of two distinct neuraminidases from avian-origin human-infecting H7N9 influenza viruses. Cell Res.

