# Supplementary material for: Highly pathogenic avian influenza H7N9 viruses with reduced susceptibility to neuraminidase inhibitors showed comparable replication capacity to their sensitive counterparts
Source: Virol J. 2019 Jul 2;16:87. doi: 10.1186/s12985-019-1194-9 (PMC6604316; doi:10.1186/s12985-019-1194-9)
Supplement: Supplementary file 1 — Table S1. Mutation on key sites of HPAI H7N9 virus from human cases. (DOCX 26 kb) [file 12985_2019_1194_MOESM1_ESM.docx]

TableS1 Mutation on key sites of HPAI H7N9 virus from human cases

| Protein | Site | Function | Amino Acid | Human Cases |
| --- | --- | --- | --- | --- |
|  |  |  |  |  |
| HA | 177^a^(186^b^) | Increased virus binding to human-type receptors. | I | 4 |
|  |  |  | **V** | 28 |
|  | 217^a^(226^b^) | Increased virus binding to human-type receptors | Q | 29 |
|  |  |  | Q\**L** | 1 |
|  |  |  | **L** | 1 |
|  |  |  | H | 1 |
| NA | 115^c^(119^d^) | Reducing the susceptibility of neuraminidase inhibitors | E | 31 |
|  |  |  | **V** | 1 |
|  | 243^c^(246^d^) |  | A | 31 |
|  |  |  | T | 1 |
|  | 271^c^(274^d^) |  | H | 31 |
|  |  |  | **Y** | 1 |
|  | 289^c^(292^d^) |  | R | 26 |
| PB2 |  | Increase the polymerase activity. | R\**K** | 1 |
|  |  |  | **K** | 5 |
|  | 526 | Enhance the 627K and 701N function. | K | 10 |
|  |  |  | **R** | 22 |
|  | 535 | Restored the polymerase activity. | M | 10 |
|  |  |  | **L** | 22 |
|  | 588 | Efficient replication in mammalian and avian cells, and higher virulence in mice | A | 20 |
|  |  |  | T | 2 |
|  |  |  | **V** | 10 |
|  | 598 | Increase the virulence in mammals. | T | 1 |
|  |  |  | **I** | 1 |
|  |  |  | V | 30 |
|  | 591 | Enhanced virulence in mice | Q | 31 |
|  |  |  | Q\**K** | 1 |
|  | 627 | Increased virulence in mammalian models. | E | 17 |
|  |  |  | E\V | 1 |
|  |  |  | E\**K** | 3 |
|  |  |  | **K** | 11 |
|  | 701 | Increased virulence in mammalian models. | D | 26 |
|  |  |  | **N** | 6 |
|  | 271 | Host signature amino acids | T | 31 |
|  |  |  | **A** | 1 |
|  | 702 |  | K | 29 |
|  |  |  | **R** | 3 |
| PA | 100 | Host signature amino acids | V | 16 |
|  |  |  | **A** | 16 |
|  | 356 |  | K | 1 |
|  |  |  | **R** | 31 |
|  | 404 |  | A | 31 |
|  |  |  | **S** | 1 |
|  | 409 |  | S | 3 |
|  |  |  | **N** | 29 |
| NP | 33 | Host signature amino acids | V | 31 |
|  |  |  | **I** | 1 |
|  | 109 |  | I | 31 |
|  |  |  | **V** | 1 |
| M1 | 41 | Impacts growth and transmission in the guinea pig model | S | 1 |
|  |  |  | **A** | 31 |
|  | 115 | Host signature amino acids | V | 31 |
|  |  |  | **I** | 1 |
| M2 | 31 | Reduced susceptibility to licensed anti-influenza medications. | **N** | 32 |
| NS1 | 42 | Altered virulence in mice. | A | 1 |
|  |  |  | **S** | 31 |
|  | 106 |  | I | 31 |
|  |  |  | **M** | 1 |
|  | PDZ ligand |  | Deletion | 30 |
|  |  |  | KPEV+7 | 1 |
|  |  |  | **ESEV** | 1 |
| NS2 | 48 | Altered antiviral response in host. | S | 1 |
|  |  |  | **A** | 31 |

a H7(H7N9)

b H3 numbering

c N9(H7N9)

d N2 numbering
